# Hybrid volumetric-modulated arc therapy for postoperative breast cancer including regional lymph nodes: the advantage of dosimetric data and safety of toxicities

**DOI:** 10.1093/jrr/rraa057

**Published:** 2020-08-12

**Authors:** Yoshiko Doi, Minoru Nakao, Hideharu Miura, Shuichi Ozawa, Masahiro Kenjo, Yasushi Nagata

**Affiliations:** 1 Hiroshima High-Precision Radiotherapy Cancer Center, 3-2-2, Futabanosato, Higashi-ku, Hiroshima-shi, Hiroshima, 732-0057, Japan; 2 Department of Radiation Oncology, Institute of Biomedical & Health Sciences, Hiroshima University, 3-2-2, Futabanosato, Higashi-ku, Hiroshima-shi, Hiroshima, 732-0057, Japan

**Keywords:** postoperative breast cancer, regional lymph nodes, hybrid volumetric-modulated arc therapy, homogeneity and conformity, lung irradiation dose

## Abstract

To improve the homogeneity and conformity of the irradiation dose for postoperative breast cancer including regional lymph nodes, we planned Hybrid volumetric-modulated arc therapy (VMAT), which combines conventional tangential field mainly for the chest area and VMAT mainly for the supraclavicular area and marginal zone. In this study, we compared the dosimetric impact between traditional 3D conformal radiotherapy (3DCRT) and Hybrid VMAT and observed toxicities following Hybrid VMAT. A total of 70 patients indicated between October 2016 and December 2017 were included. The prescribed dose was 50 Gy/25 fractions. For the dosimetric impact, 3DCRT and Hybrid VMAT plans were compared in each patient with respect to the dosimetric parameters. Toxicities were followed using the Common Terminology Criteria for Adverse Events version 4.0. The median follow-up duration was 319 days. For the dosimetric impact, the homogeneity index (HI) and conformity index (CI) of PTV were significantly improved in the Hybrid VMAT plan compared with that in the 3DCRT plan (HI, 0.15 ± 0.07 in Hybrid VMAT vs 0.41 ± 0.19 in 3DCRT, *P* < 0.001; CI, 1.61 ± 0.44 in Hybrid VMAT vs 2.10 ± 0.56 in 3DCRT, *P* < 0.001). The mean irradiated ipsilateral lung dose was not significantly different in both plans (12.0 ± 2.4 Gy in Hybrid VMAT vs 11.8 ± 2.8 Gy in 3DCRT, *P* < 0.533). Regarding toxicity, there were no patients who developed ≥grade 3 acute toxicity and ≥grade 2 pneumonitis during the follow-up. Hybrid VMAT for postoperative breast cancer including regional lymph nodes was a reasonable technique that improved the homogeneity and conformity of the irradiation dose to the planning target volume while keeping the irradiation dose to organs at risk to a minimum.

## INTRODUCTION

Radiotherapy (RT) as the management strategy of breast cancer is an essential local therapy to reduce locoregional recurrence and improve survival after not only breast-conserving surgery but also radical mastectomy [1–3]. Conventionally, when we prescribed RT including regional lymph nodes for breast cancer, 3D conformal RT (3DCRT) was the adopted approach, with tangential fields for the chest area and separate fields for supraclavicular nodes. In fact, although tangential beam orientation is optimal for limiting low doses to normal tissues, traditional 3DCRT plans provide inadequate nodal coverage, and the conformity of dose distributions is relatively poor [4]. To obtain a high improvement in homogeneity and conformity for the targets while maintaining a low irradiation dose to the normal tissues, we devised an original technique, which combined conventional tangential field mainly for the chest area and volumetric-modulated arc therapy (VMAT) mainly for the supraclavicular area and marginal zone. This technique was named ‘Hybrid VMAT’. Since October 2016, all patients who were determined to require radiation including regional lymph nodes after breast cancer surgery in October 2016 received the Hybrid VMAT technique as a general rule. In this study, we compared the dosimetric impact between traditional 3DCRT and Hybrid VMAT and observed toxicities following Hybrid VMAT.

**Fig. 1.**
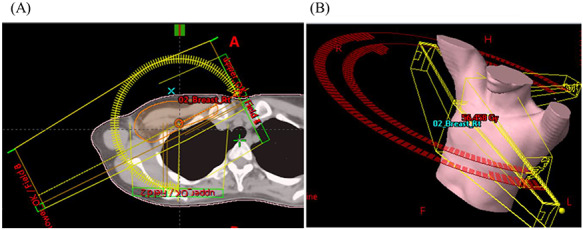
Beam arrangement of H-VMAT. (**A**) Axial CT image and (**B**) overhead view of beam setup. Hybrid VMAT (H-VMAT) combines conventional tangential field mainly for the chest area and VMAT mainly for the supraclavicular area and marginal zone.

## MATERIALS AND METHODS

### Patient data

This study included all cases requiring radiation including regional lymph nodes after breast cancer surgery between October 2016 and December 2017. Adjuvant RT including regional lymph nodes after surgery for breast cancer was adapted based on the guidelines for breast cancer in Japan [5] and the results of consultation with attending physicians.

All patients provided written informed consent for their clinical data to be included in the study analyses prior to treatment. The study was approved by our institutional review board.

This study has two parts. The first part discusses the dosimetric characteristics between traditional 3DCRT simulation and Hybrid VMAT. The second part evaluates toxicities following Hybrid VMAT.

### Contour definition

Clinical target volume (CTV) was defined as the entire ipsilateral chest area with supraclavicular nodes based on not only the Radiation Therapy Oncology Group (RTOG) Breast Cancer Atlas but also clinical data for each patient. We also confirmed that the results of contouring the CTV did not exceed the range of conventional 3DCRT. The planning target volume (PTV) was obtained by adding a 5-mm margin around the CTV. The radiation oncologist then manually modified the shape of the PTV to make it smooth. The PTV-mo for evaluation was derived from the PTV with a 2-mm margin on the skin surface and lung around the PTV. The organs at risk (OARs) surrounding the targets, including bilateral lungs, heart, contralateral breast, ipsilateral humeral head, spinal cord and esophagus, were also contoured. All processes of delineation were checked by radiation oncologists.

### 3DCRT simulation planning

For each patient, traditional 3DCRT and Hybrid VMAT plans were generated. A 3DCRT plan, only for the simulation study, was designed with four fields, with two main tangential fields for chest area and two anterior–posterior fields for supraclavicular nodes traditionally. Field-in-field techniques were also used, if necessary. Each field for the chest area was determined using the field technique to ensure that the Dmax of PTV was <110% of the prescribed dose.

### Hybrid VMAT planning

Figure 1 shows the beam arrangement of the Hybrid VMAT plan. First we set the isocenter point to the chest, which is the level 2 cm caudal to the upper sternum, and calculated the main tangential fields for the chest area. Field-in-field techniques were also used if necessary. Then we extracted a marginal zone with insufficient dose (≤95% of prescription dose) by tangential irradiation, and optimized the VMAT plan for the supraclavicular area and marginal zone based on the results of calculation of the tangential fields for the chest area. The marginal zone is usually confined to a narrow area on the thoracic side and does not cover the entire chest area.

**Fig. 2.**
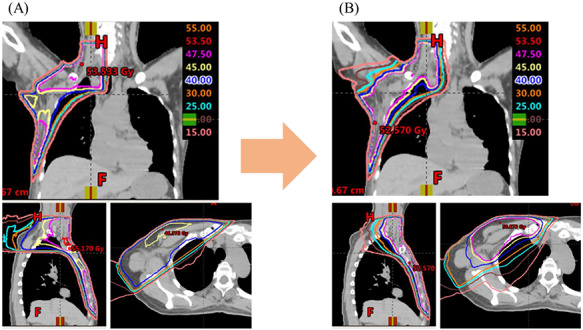
Comparison of distribution maps. (**A**) Axial CT image and (**B**) overhead view of beam setup. ‘Hybrid VMAT (H-VMAT)’ combines conventional tangential field mainly for the chest area and VMAT mainly for the supraclavicular area and marginal zone.

The VMAT plan generated two coplanar arcs (one counterclockwise and another clockwise) with gantry rotation angles of 240° (ranging from 60 to 181° and 181 to 60° for right-sided primary tumors and from 179 to 300° and 300 to 179° for left-sided primary tumors). The collimator angle of each arc was set to 10 or 80° to avoid a tongue-and-groove effect. The details of OAR and PTV dose constraints are shown in Table 1. Figure 2 shows the comparison of distribution maps between conventional 3DCRT and Hybrid VMAT.

**Table 1:** Dose constraints for Hybrid VMAT optimization

		Constraint	Preferable
PTV-mo	D50	97–99%	-
	Max	≤115%	≤120%
	D95	92–96%	
Esophagus	D1cc	≤25 Gy	≤20 Gy
	D5cc	≤20 Gy	≤15 Gy
Lungs	V5	≤35%	≤30%
	Mean	≤10 Gy	≤8 Gy

All radiation plans in this study were performed in the Eclipse treatment planning system (Varian Medical Systems, Palo Alto, CA, PRO, AcurosXB), using 6-MV photons generated by a linear accelerator (TrueBeam, Varian Medical Systems). To ensure accurate delivery of each Hybrid VMAT plan, daily bone matching was performed using orthogonal kV electronic portal images and digitally reconstructed radiographs, which were created from planning computed tomography (CT) data. We also acquired kV cone-beam CT (CBCT) images after bone matching 2 or 3 days from the initiation of treatment and weekly thereafter to verify patient position.

### Plan evaluation and statistical analysis

 In the dosimetric analysis, the following indices extracted from dose–volume histograms were used:

(i) maximum dose (Max), minimum dose (Min), homogeneity index (HI) and conformity index (CI) for PTV. HI and CI were calculated according to the definition proposed by the International Commission on Radiation Units and Measurements [6] and expressed as follows:

(10) $$ {\rm{HI}}=\frac{D_{2\%}-D_{98\%}}{D_{50\%}}\qquad{\rm{CI}}= \frac{TV_{\geq 95\%}}{PTV_{\geq 95\%}} $$

(ii) irradiation dose to OARs, such as the lung (ipsilateral lung V5Gy, V20Gy and mean dose, and contralateral lung V5Gy and mean dose), heart (D2cc) and esophagus (D5cc).

We applied box-and-whisker plots and the Wilcoxon signed-rank test to compare irradiation doses during 3DCRT and Hybrid VMAT. *P*-values < 0.05 were considered statistically significant.

### Clinical study

The prescription dose for all patients was 50 Gy in 25 fractions to conserved breast or chest wall and regional lymph nodes using 6-MV photon. When the surgical margin was close to the primary tumor for breast-conserving surgery, boost irradiation of 10 Gy in 5 fractions was added to the original tumor bed using electrons with energy of 6–12 MeV. In patients undergoing radical mastectomy, a 0.5-cm-thick solid bolus was added to the chest wall daily in the latter half of the radiation therphy period to increase the skin dose.

Each patient was regularly followed by the treating physician twice a week during RT. Radiation dermatitis (RD) was checked up to 1 month after treatment completion. The time of occurrence and site of the most severe RD were recorded for each patient. Radiation pneumonitis (RP) was assessed within 6 months to 1 year. All acute side effects were graded according to the Common Terminology Criteria for Adverse Events (version 4.0) issued by the National Cancer Institute.

## RESULTS

### Clinical characteristics

Two patients were male and 68 patients were female. There were 35 patients with left-sided and 35 patients with right-sided tumor. The median age of the patients was 56 years (range, 26–81 years). A total of 19 patients underwent breast-conserving surgery, 48 patients underwent radical mastectomy and 3 patients underwent radical mastectomy for postoperative local recurrence. Of 12 patients with cN0 disease, nine were indicated because they had pN1 or higher. The remaining three cN0 patients had large tumors; therefore, regional lymph nodes were included in consultation with the attending physician. One patient had pTisN2b disease, a very rare condition; therefore, regional lymph nodes were included in consultation with the attending physician. A total of 64 patients underwent axillary lymph node dissections and 6 patients underwent only sentinel axillary lymph node biopsies. These 6 patients were considered clinical complete response (cCR) because of neoadjuvant preoperative pharmaceutical therapy. Fifty patients received neoadjuvant preoperative pharmaceutical therapy. Twelve patients received chemotherapy including anti-HER2 drugs, 7 patients received chemotherapy including anti-vascular endothelial growth factor drugs and 3 patients received hormonal therapy; the remaining 28 patients received only chemotherapy. Four cases were clinically positive for internal mammary lymph nodes. No cases had pathologically confirmed internal mammary lymph nodes. Table 2 shows the other patient characteristics.

**Table 2:** Patient characteristics

Variables	Units	Number
Sex	Male/female	2/68
Age (years)	Median (range)	56 (26–81)
Location	Left/right	35/35
Surgery technique	BC^a^/MA^b^	19/51
Histology	Non-IDC^c^/Invasive ductal carcinoma (IDC)/Invasive lobular carcinoma (ILC)/other	1/60/7/2
Stage (clinical)	T1a/T1c/T2/T3/T4b	1/15/31/10/13
	N0/N1/N2a/N2b/N3a/N3b/N3c	15/26/17/2/4/3/3
Neoadjuvant pharmaceutical therapy	Yes/no	50/20
Histological therapeutic effect	G1a/G1b/G2a/G2b/G3	13/10/6/5/14
Adjuvant chemotherapy	Yes/no	36/44

^a^Breast-conserving surgery; ^b^mastectomyS; ^c^pTisN2b, a very rare condition.

### Comparison of dosimetric parameters of the PTV-mo

**Fig. 3.**
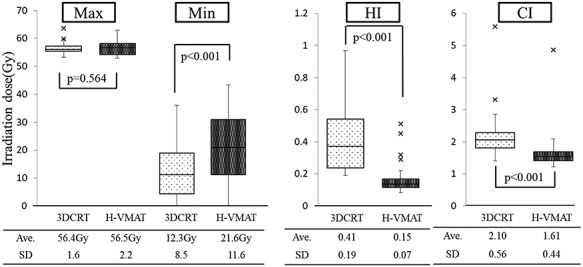
Comparison of dosimetric parameters: PTV-mo. Box-and-whisker plots of the irradiation dose of PTV-mo are shown. The band inside the boxes represents the median, and the bottom and top of the boxes represent the 25th and 75th percentiles, respectively. The whiskers indicate the lowest datum still within the 1.5 interquartile range (IQR) of the lower quartile, and the highest datum still within the 1.5 IQR of the upper quartile. Crosses (‘x’) indicate outliners of maximum displacement. The maximum (Max) of PTV-mo was almost similar in both plans. The minimum (Min) of PTV-mo was significantly higher in the Hybrid VMAT plan compared with that in the 3DCRT plan. Homogeneity index (HI) and conformity index (CI) were significantly improved for the Hybrid VMAT plan compared with the 3DCRT plan. Ave = average, SD = standard deviation.

The maximum PTV-mo was almost similar in both plans (average 56.4Gy ± 1.6 for the 3DCRT plan vs average 56.5Gy ± 2.2 for the Hybrid VMAT plan, *P* = 0.564). The minimum PTV-mo was significantly higher in the Hybrid VMAT plan compared with that in the 3DCRT plan (average 12.3Gy ± 8.5 for 3DCRT plan vs average 21.6Gy ± 11.6 for the Hybrid VMAT plan, *P* < 0.001). HI and CI were significantly improved for the Hybrid VMAT plan compared with the 3DCRT plan (HI, average 0.41 ± 0.19 for 3DCRT plan vs average 0.15 ± 0.07 for the Hybrid VMAT plan, *P* < 0.001; CI, average 2.10 ± 0.56 for the 3DCRT plan vs average 1.61 ± 0.44 for the Hybrid VMAT plan, *P* < 0.001). Figure 3 shows the dosimetric parameters of PTV for Hybrid VMAT and 3DCRT.

### Comparison of dosimetric parameters of the lungs

**Fig. 4.**
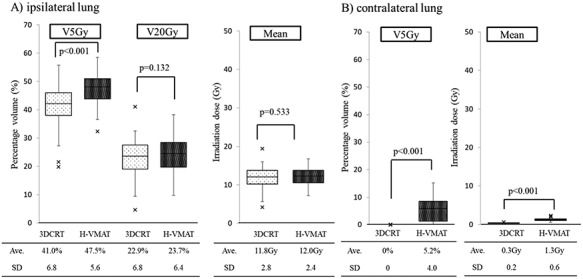
Comparison of dosimetric parameter: lungs. Box-and-whisker plots of the dosimetric parameters of lungs are shown. The values of the dosimetric parameters V5 of the ipsilateral lung was significantly higher in the Hybrid VMAT plan compared with those in the 3DCRT plan. The values of the dosimetric parameters V20 and mean irradiated dose of the ipsilateral lung were not significantly different between the Hybrid VMAT and 3DCRT plans. The values of the dosimetric parameters V5 and mean irradiated dose of the contralateral lung were significantly higher in the Hybrid VMAT plan compared with those in the 3DCRT plan.

The values of the dosimetric parameter V5 of the ipsilateral lung was significantly higher in the Hybrid VMAT plan compared with those in the 3DCRT plan (average 41.0% ± 6.8 for the 3DCRT plan vs average 47.5% ± 5.6 for the Hybrid VMAT plan, *P* < 0.001). The values of the dosimetric parameter V20 and mean irradiated dose of the ipsilateral lung were not significantly different between the Hybrid VMAT and 3DCRT plans (V20, average 22.9% ± 6.8 for 3DCRT plan vs average 23.7% ± 6.4 for Hybrid VMAT plan, *P* = 0.132; mean irradiated dose, 11.8Gy ± 2.8 for the 3DCRT plan vs average 12.0 Gy ± 2.4 for the Hybrid VMAT plan, *P* = 0.533). The values of the dosimetric parameter V5 and mean irradiated dose of the contralateral lung were significantly higher in the Hybrid VMAT plan compared with those in the 3DCRT plan (V5, average 0% for the 3DCRT plan vs average 5.2% ± 4.0 for the Hybrid VMAT plan, *P* < 0.001; mean irradiated dose, 0.3 Gy ± 0.2 for the 3DCRT plan vs average 1.3Gy ± 0.6 for the Hybrid VMAT plan, *P* < 0.001). Figure 4 shows the dosimetric parameters of ipsilateral lung (Fig. 4A) and contralateral lung (Fig. 4B) for Hybrid VMAT and 3DCRT.

### Comparison of dosimetric parameters of the heart and esophagus

**Fig. 5.**
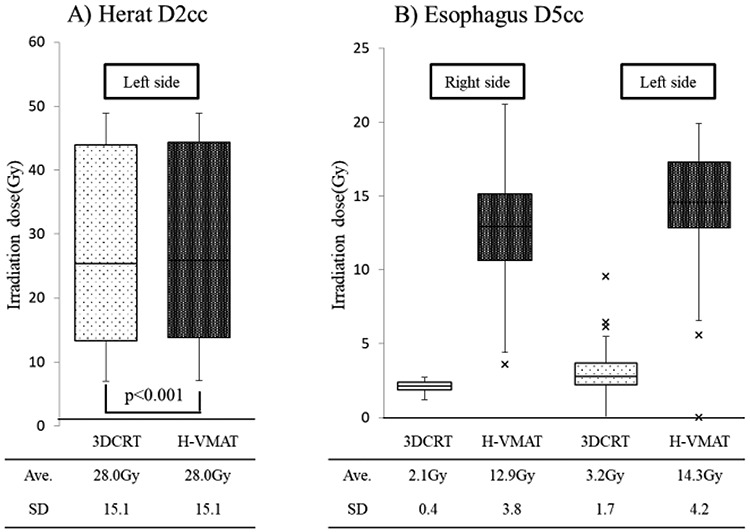
Comparison of dosimetric parameters: heart and esophagus. Box-and-whisker plots of the dosimetric parameters of heart and esophagus are shown. The mean irradiated heart dose for patients with left-sided tumor was almost similar in both plans. The D5cc dose of the esophagus was significantly higher in the Hybrid VMAT plan compared with that in the 3DCRT plan.

The mean irradiated heart dose for patients with left-sided tumor was almost similar in both plans (11.8Gy ± 2.8 for the 3DCRT plan vs average 12.0 Gy ± 2.4 for the Hybrid VMAT plan, *P* < 0.001). The D5cc dose of the esophagus was significantly higher in the Hybrid VMAT plan compared with that in the 3DCRT plan (patients with right-sided tumor, 2.1 Gy ± 0.4 for the 3DCRT plan vs average 12.9 Gy ± 3.8 for the Hybrid VMAT plan, *P* < 0.001; patients with left-sided tumor, 3.2 Gy ± 1.7 for the 3DCRT plan vs average 14.3 Gy ± 4.2 for the Hybrid VMAT plan, *P* < 0.001). Figure 5 shows the dosimetric parameters of heart D2cc (Fig. 5A) and esophagus D5cc (Fig. 5B) for Hybrid VMAT and 3DCRT.

### Evaluation of toxicities

The median follow-up duration was 319 days. A total of 31 (44%) and 39 (56%) patients had ≥grade 1 and ≥grade 2 RD, respectively. In patients who developed ≥grade 2 RD, 30 patients underwent mastectomy and the other 9 patients underwent breast-conserving surgery. There were no patients with ≥grade 3 RD. Eleven (16%) patients (left side, 7 patients; right side, 4 patients) had ≥grade 1 radiation esophagitis. Fifty-nine patients (84%) did not have discomfort during swallowing. Only one patient developed ≥grade 1 RP during the follow-up. Almost all patients did not have RP. Table 3 shows the evaluation of toxicity.

**Table 3:** Evaluation of toxicity

	Grade 0	Grade 1	Grade 2	≥Grade 3
Dermatitis	0 (0%)	31 (44%)	39 (56%) Chest wall 30 patients; conserved breast 9 patients	0 (0%)
Esophagitis	59 (84%)	11 (16%) Left side 7 patients; right side 4 patients	0 (0%)	0 (0%)
Pneumonitis	69 (99%)	1 (1%)	0 (0%)	0 (0%)

## DISCUSSION

We devised the original technique ‘Hybrid VMAT’, which combines conventional tangential field mainly for chest area and VMAT mainly for supraclavicular area and marginal zone. Our method differs from the method commonly called the Hybrid technique, which was a combination of Intensity modulated radiation therapy (IMRT)/VMAT and 3DCRT [7–13, 19]. Many studies have performed simulation studies on breast or chest wall targets. We performed a unique method targeting the breast or chest wall, including the regional area, and confirmed its safety. We believe our method has two advantages. First, we can achieve the minimum low dose to OARs, especially the lung, using our Hybrid VMAT technique. When good PTV coverage is needed using general IMRT/VMAT, an increase in low-dose irradiation area to OARs is unavoidable compared with the result of the conventional 3DCRT plan. In particular, it is easy to expand the low-dose area to the lung. There have been several studies on IMRT/VMAT planning for postoperative breast cancer including regional lymph nodes, and the irradiation dose to the lung in other published studies is shown in Table 4 [14–19]. In these studies, the summarized V5 and V20 of the ipsilateral lung were 51–99.3% and 17.7–32.3%, respectively. Relatively high V5 and V20 of the lung were accepted in those studies. However, it is important to improve the coverage of PTV without increasing the dose to the OARs, even if using IMRT/VMAT. In particular, we recognize that increasing the irradiation dose to the lung may increase the risk of RP and should be given cautiously. In our results with Hybrid VMAT, the V5, V20 and mean irradiated doses of the ipsilateral lung were 47.5% ± 5.6, 23.7% ± 6.4 and 12.0 Gy ± 2.4, respectively. It was found that this result did not differ greatly from the result with 3DCRT. Clinical results showed no appearance of RP with clinical symptoms. Unfortunately, as a result of reducing the dose to the lung field, the irradiation dose to the esophagus increased slightly compared with 3DCRT. However, in clinical studies, esophagitis has no effect on eating disorders. We recognized that an increase in irradiation dose to the esophagus by our method is clinically acceptable. Our method has come closer to the ideal approach that improves the homogeneity and conformity of the irradiation dose to PTV while keeping the irradiation dose to the OARs to a minimum.

**Table 4:** Comparison of irradiation lung dose

	Ipsilateral lung dose			Contralateral lung			Double lungs		
	V5	V20	Dmean	V5	V20	Dmean	V5	V20	Dmean
Ma *et al*. [14]	65% ± 8	28% ± 2	15.06 Gy ± 1.66	12% ± 11	-	2.26 Gy ± 1.32	-	-	-
Nicols *et al*. [15]	VMAT: 96.9% ± 1.3	VMAT: 32.3% ± 0.8	-	VMAT: 37.8% ± 4.9	-	-	VMAT: 66.2% ± 3.1	VMAT: 16.3% ± 0.2	-
	HT (Helical TomoTherapy): 99.3% ± 0.5	HT :29.9% ± 1.0		HT :81.7% ± 2.5			HT :89.8% ± 1.4	HT :15.0% ± 0.3	
Zhao *et al*. [16]	65.2% ± 6.7	17.7% ± 4.1	7.9 Gy ± 2.2	-	-	-	43.5% ± 6.7	10.3% ± 5.7	6.5 Gy ± 16.6
Lai *et al*. [17]	70.3% ± 5.8	23.1% ± 2.3	13.5 Gy ± 0.6	44.5% ± 6.5	1.3% ± 1.0	5.1 Gy ± 0.7	-	-	-
Boman *et al*. [18]	58–87%	26–37%	14.4 Gy–18.6 Gy	3.0–27.7%	-	0.7 Gy–4.1 Gy	-	-	-
Balaji *et al*. [19]	51–57%	23–24%	12.7 Gy–14.3 Gy	0.07–11.9%	-	-	-	-	-
This study	47.5% ±5.6	23.7% ± 6.4	12.0 Gy ±2.4	5.2% ±4.0	0	1.3 Gy ± 0.6	26.4% ± 4.8	11.8% ± 3.3	6.7 Gy ± 3.8

Second, there was no irradiation to the contralateral chest area using our Hybrid VMAT technique because we used only the conventional tangential field for chest area PTV. If there is a history of breast cancer treatment, the rate of developing breast cancer in the contralateral breast is relatively high. When radiation to the contralateral side is needed, we have to be more careful not to overdose around the middle of the chest. In our Hybrid VMAT technique, the irradiated dose was not spread to the contralateral sides because we only used tangential beam for the conserved breast or chest. The treatment plan using IMRT/VMAT might become complex because IMRT/VMAT for the breast easily expands the irradiation dose to the contralateral side. We consider that a flexible technique that can handle any situation is required for patients with breast cancer, and our Hybrid VMAT technique is the best method.

Fixed accuracy is important in IMRT/VMAT. The main uncertainties in breast treatment are the setup and respiratory motion [20, 21]. To reduce the setup uncertainty, a sufficient target expansion margin and daily image guidance should be considered. We have kept in mind that there was enough margin when setting up the PTV and we have also verified patient position using CBCT images. Moreover, our technique is less susceptible to respiratory motion because we used only open fields for the chest area. We consider our method to be a robust method for setup and respiratory motion.

We recognized that more detailed research and analysis are required among various hybrid techniques that include different VMAT designs for further improvement in plan quality. In particular, patients with breast cancer achieved long-term survival even in the advanced stage. Even if our method does not increase low dose irradiation to OARs much, we should always be careful about secondary carcinogenesis. In some literature, the increase in low-dose irradiation volume to OARs raises the concern of radiation-induced secondary cancers [22, 23]. Thus, we recognize that there is a significant clinical need to reduce low irradiation doses as much as possible. Additionally, prospective clinical studies with long-term follow-up in patients treated with Hybrid techniques are needed.

## CONCLUSION

Our Hybrid VMAT technique for postoperative breast cancer including regional lymph nodes was a reasonable technique that improved the homogeneity and conformity of the irradiation dose to PTV while keeping the irradiation dose to the OARs to a minimum. Furthermore, our Hybrid VMAT was proved to be a safe technique in the evaluation of toxicity.
